# Digital health literacy and sports participation among college students: the role of cognitive flexibility and physical activity self-efficacy

**DOI:** 10.3389/fpsyg.2026.1921192

**Published:** 2026-09-18

**Authors:** Yuming Xu, Jun Peng, Yuting Xu, Nuan Wen

**Affiliations:** 1 School of Physical Education, South China University of Technology, Guangzhou, China; 2 Faculty of Teacher Education, Ningbo University, Ningbo, China; 3 Faculty of Education, City University of Macau, Macau, Macao SAR, China

**Keywords:** cognitive flexibility, college students, digital health literacy, physical activity, self-efficacy, sports participation

## Abstract

**Background:**

Digital health literacy is increasingly relevant to health behavior among college students, but its relationships with sports participation and associated psychological factors remain incompletely understood. Guided by social cognitive theory and an adaptive cognitive-processing perspective, this study examined a prespecified cross-sectional path model involving digital health literacy, cognitive flexibility, physical activity self-efficacy, and sports participation.

**Methods:**

A cross-sectional survey was conducted among 600 Chinese college students. Participants completed the DHLI 2.0, CFI, PASE, and PARS-3. Pearson correlation analysis, structural equation modeling, and bootstrap estimation of model-specific path-coefficient products using 5,000 resamples were performed with SPSS 26.0 and AMOS.

**Results:**

Digital health literacy was positively associated with cognitive flexibility, physical activity self-efficacy, and sports participation (all *p* < 0.001). The structural model demonstrated a good fit to the data (*χ*^2^/df = 1.984, RMSEA = 0.016, SRMR = 0.033, CFI = 0.986, TLI = 0.993). Within the prespecified model, digital health literacy was positively associated with cognitive flexibility (*β* = 0.504), physical activity self-efficacy (*β* = 0.213), and sports participation (*β* = 0.191). The three-path coefficient product was small, although its bootstrap confidence interval excluded zero (standardized estimate = 0.011, 95% CI [0.002, 0.031]).

**Conclusion:**

The observed covariance pattern was consistent with the prespecified cross-sectional path model involving cognitive flexibility and physical activity self-efficacy. However, because all variables were measured at a single time point, the findings do not establish temporal ordering or causal mediation. Longitudinal and experimental studies are needed to examine temporal ordering and causal relationships among these variables.

## 1 Introduction

Digital technology is profoundly changing the health behaviors of college students (Jiang et al., 2024). The World Health Organization reports a widespread decline in physical activity among adolescents and young adults globally, with sedentary behavior and digital dependence becoming significant health risks (Bull et al., 2020). Insufficient physical activity remains a prominent issue among college students. With the rapid popularization of mobile internet, smart wearable devices, and online fitness platforms, an increasing number of university students are accessing health and exercise information through digital media, such as using fitness apps to record exercise data, learning fitness techniques through short video platforms, or participating in online sports interactions via social media (Cho et al., 2014; Singh et al., 2025). Against this backdrop, digital health literacy is gradually becoming an important factor in promoting healthy behaviors among young people (Ridwan et al., 2022; Zhu et al., 2024). Existing research shows that digital health literacy not only relates to an individual’s ability to access and understand health information but also further influences their health decisions and behaviors (Dadaczynski et al., 2021; Norman and Skinner, 2006). For college students, higher digital health literacy can help them more effectively filter exercise information, discern the authenticity of online health content, and enhance their initiative in participating in physical activities (Bayati et al., 2018).

The relationship between digital health literacy and health promotion has received widespread attention (Liu H. X. et al., 2024). Studies have found significant associations between digital health literacy and physical activity, healthy lifestyles, and mental health (Kühn et al., 2022; Li and Yang, 2024). Particularly among college students, individuals with higher digital health literacy tend to develop positive health behavior patterns, including regular exercise, healthy eating, and proactive health management (Paige et al., 2018). However, existing research focuses primarily on the direct impact of digital health literacy on general health behaviors, lacking in-depth exploration of the underlying psychological mechanisms by which it affects college students’ participation in sports. In fact, whether college students truly participate in sports depends not only on their ability to acquire sports knowledge but also on their cognitive processing abilities and behavioral beliefs (Acar et al., 2024). As defined by Berkman and colleagues, health literacy (HL) is “the degree to which individuals can obtain, process, understand, and communicate about health-related information needed to make informed health decisions” (Berkman et al., 2010). Therefore, explaining the impact of digital health literacy on sports participation solely from the perspective of “information acquisition” remains limited.

First proposed in cognitive psychology, cognitive flexibility emphasizes an individual’s ability to adjust cognitive strategies, shift thinking patterns, and adapt to new situations in complex environments (Dennis and Vander Wal, 2010). In a digital environment, individuals are bombarded with a vast amount of health and exercise information daily. A high level of digital health literacy can help college students process complex information more effectively and enhance their cognitive flexibility (Hohl and Dolcos, 2024; Liu L. et al., 2024). Existing research has shown a significant association between cognitive flexibility and health behavior, self-regulation, and proactive decision-making (Martin and Rubin, 1995; Tong et al., 2023). College students with higher cognitive flexibility are more likely to adjust their exercise plans, overcome barriers to exercise, and try different forms of physical activity (Shokrkon and Nicoladis, 2022; Tong et al., 2023). Accordingly, cognitive flexibility was examined as a possible statistical link between digital health literacy and sports participation.

Furthermore, physical activity self-efficacy is also an important psychological factor influencing college students’ sports participation. Based on Bandura’s social cognitive theory, self-efficacy refers to an individual’s confidence in their ability to perform a certain behavior (Bandura, 2013). In the field of sports, physical activity self-efficacy is mainly reflected in an individual’s belief that they can persevere in exercising even when faced with situations such as insufficient time, academic pressure, or physical fatigue. Numerous studies have confirmed a significant positive correlation between physical activity self-efficacy and sports participation (Dishman et al., 2005; Shudayfat et al., 2023). Meanwhile, individuals with higher cognitive flexibility typically possess stronger problem-solving and behavioral regulation abilities, which contribute to increased control and confidence (Zhang et al., 2026). Self-efficacy is recognized as an important component of self-management and a key factor in promoting behavioral change and maintaining optimal functioning (Huang et al., 2026). In the present model, physical activity self-efficacy was therefore positioned as the more proximal correlate of sports participation.

Existing research consistently demonstrates that cognitive flexibility enhances an individual’s sense of behavioral control and self-regulation, factors closely related to the formation of self-efficacy. A study by Bebeley et al. (2017) in a university student population found that exercise self-efficacy plays a significant mediating role between exercise motivation and physical activity, indicating that an individual’s perception of their own exercise capabilities further influences actual exercise behavior. Similarly, Mohammadkhani et al. (2022) also pointed out that cognitive flexibility can further promote positive behavioral performance by improving an individual’s psychological adaptability.

Behavioral participation depends not only on the acquisition of external information but also on an individual’s cognitive interpretation and self-regulatory beliefs (Bandura, 2013). Digital health literacy can first serve as a cognitive resource, enabling individuals to assess, organize, and adaptively process digital health information. These associations may be reciprocal, and physical activity itself may strengthen self-efficacy or cognitive flexibility. The proposed order should therefore be understood as a theory-informed specification rather than an empirically established temporal process.

Social cognitive theory provides the basis for treating physical activity self-efficacy as a behavior-specific belief closely associated with participation (Bandura, 2013). The adaptive cognitive-processing perspective, informed by the CFI framework, supports examining cognitive flexibility as a general capacity for generating alternatives and perceiving control (Dennis and Vander Wal, 2010). Neither framework directly specifies that digital health literacy must precede cognitive flexibility, that ordering is an integrative extension proposed in this study.

Digital health literacy was conceptualized as a domain-specific informational resource, cognitive flexibility as a broader adaptive processing tendency, physical activity self-efficacy as a behavior-specific belief, and sports participation as the behavioral outcome. This ordering is plausible, but alternatives are also reasonable. Cognitively flexible students may navigate digital information more effectively, and physically active students may gain both efficacy and digital health experience (Woo et al., 2024; Yu et al., 2023). Therefore, the present study proposes a sequential “cognition-belief-behavior” pathway to explain how digital health literacy may relate to sports participation among college students.

## 2 Theoretical framework and hypotheses

Although prior research has examined pairwise associations among digital health literacy, cognitive flexibility, physical activity self-efficacy, and sports participation, evidence integrating all four constructs remains limited. Therefore, this study evaluated hypothesized indirect association models. The association between digital health literacy and sports participation was examined, and specific indirect associations were estimated through cognitive flexibility and physical activity self-efficacy.

Building on the findings of previous research, the following hypotheses are proposed for this study (See Figure 1):

**Figure 1:**
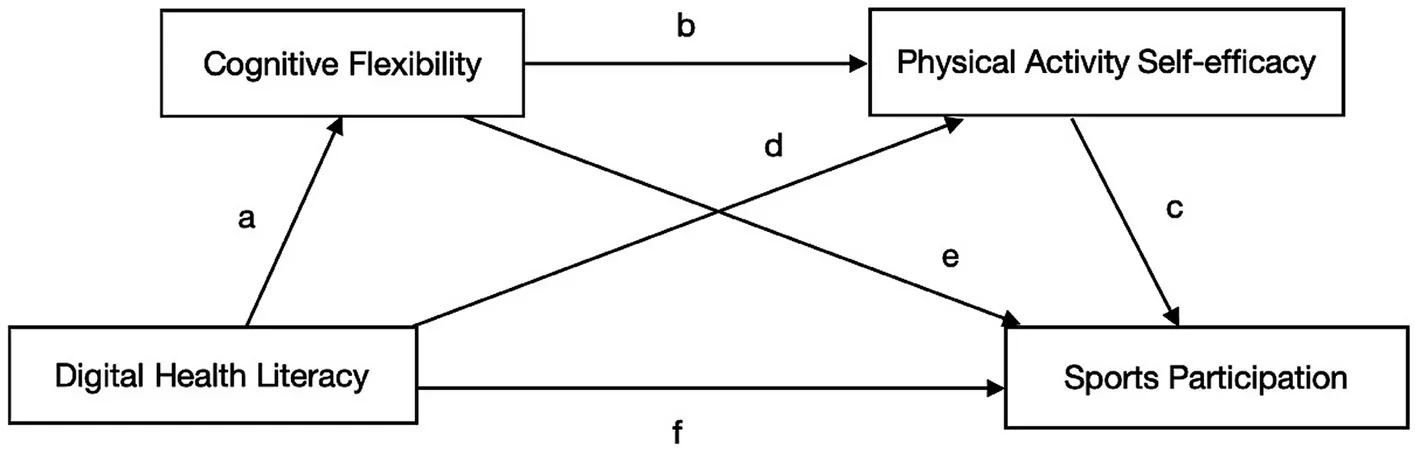
Hypothetical model.

*H1:* Digital health literacy is positively associated with sports participation.

*H2:* Digital health literacy has a positive cross-sectional indirect association with sports participation through cognitive flexibility.

*H3:* Digital health literacy has a positive cross-sectional indirect association with sports participation through physical activity self-efficacy.

*H4:* Digital health literacy has a positive ordered cross-sectional indirect association with sports participation through cognitive flexibility and then physical activity self-efficacy.

## 3 Methods and measures

### 3.1 Participants

All data were collected in May 2026 through a combination of online and offline methods. Participants were recruited from Guangdong University of Finance and Economics. To improve the representativeness of the sample and ensure coverage of college students at different learning stages, this study employed a stratified random sampling method based on grade level, selecting college students from four grades as research subjects and distributing a total of 650 questionnaires. Eligibility criteria included being a current undergraduate student, aged 18 or above, able to read Chinese, and providing informed consent. Of the 639 returned questionnaires, 39 were excluded. These included nine questionnaires with missing answers to core scale items, 17 questionnaires with response times significantly shorter than reasonable completion times, and 13 questionnaires with obvious patterns or inconsistencies in the responses. The final analysis sample consisted of 600 participants (valid response rate = 93.9%). Participants included 296 males (49.33%) and 304 females (50.67%); 309 from rural areas and 291 from urban areas. The grade distribution was as follows: 149 first-year college students (24.83%), 148 s-year college students (24.67%), 150 third-year college students (25.00%), and 153 fourth-year college students (25.50%). The study was approved by the Guangdong University of Finance and Economics ethics committee (GDUFE202109-01) and conducted in accordance with the Declaration of Helsinki. Before beginning, participants received information about the study purpose, procedures, voluntary participation, anonymity, and right to discontinue. Consent was indicated before questionnaire completion; the ethics approval did not require a signed consent form for this anonymous, minimal-risk survey.

### 3.2 Measures

This study included demographic components (including subject, gender, region, and grade) and four standardized questionnaires to measure digital health literacy, cognitive flexibility, self-efficacy, and sports participation: the Digital Health Literacy Questionnaire (DHLI), the Cognitive Flexibility Inventory (CFI), the Physical Activity Self-Efficacy Scale (PASE), and the Physical Activity Rating Scale-3 (PARS-3). All questionnaires were previously drafted and validated in English. In this study, all questionnaires were translated from English to Chinese by a bilingual expert in sports psychology and from Chinese to English by a Chinese expert in English linguistics. The two experts then agreed on the final Chinese version of the questionnaire items in a discussion. This Chinese translation process followed the Brislin Standard for forward-backward translation (Brislin, 1970).

#### 3.2.1 Digital Health Literacy Instrument 2.0 Short Form

This survey tool was originally developed by van der Vaart and Drossaert (2017) using the Digital Health Literacy Tool 2.0 Short Form (DHLI 2.0). This study uses an updated version of the Digital Health Literacy Tool (DHLI 2.0) short form. The questionnaire contains 16 items across 8 dimensions: Operational skills (e.g., “Use the buttons and links or hyperlinks on websites?” easy or difficult), Navigation skills (e.g., “You lose track of where you are on a website or in an app?” how often), Information searching (e.g., “Find exactly what you are looking for?” easy or difficult), Evaluating reliability (e.g., “Determine whether the information is reliable or not?” easy or difficult), Determining relevance (e.g., “Determine whether the information found applies to you?” easy or difficult), Generating content (e.g., “Express your opinions, thoughts, or feelings in writing?” easy or difficult), Using security measures (e.g., “Create and remember strong passwords?” easy or difficult) and Protecting privacy (e.g., “do you include sensitive personal information in your message?” How often). Each item is rated on a 4-point scale (“Very difficult” = 1 to “Very easy” = 4), with all responses converted so that higher scores reflect higher levels of digital health literacy. For the present structural model, all 16 items were specified as indicators of one overarching digital health literacy factor because the hypotheses concerned overall digital health literacy. The measurement tool demonstrated good reliability and validity, with a Cronbach’s alpha of 0.867, which meets the standard of greater than 0.7.

#### 3.2.2 The Cognitive Flexibility Inventory

The Cognitive Flexibility Scale (CFI) was originally developed and validated by Dennis and Vander Wal (2010) to measure the type of cognitive flexibility necessary for individuals to successfully challenge and replace maladaptive thoughts with more balanced and adaptive thinking (Dennis and Vander Wal, 2010). The scale consists of 20 items, comprising two dimensions: Alternatives and Control subscales. A 7-point Likert scale was used for scoring, ranging from “Strongly Disagree = 1” to “Strongly Agree = 7”. After back-scoring, the scores of all items were summed to obtain the total score. A higher total score indicates a higher level of cognitive flexibility. Because the structural hypothesis concerned overall cognitive flexibility, the present study specified the 20 items as indicators of one overarching latent factor. The CFI has a reliable two-factor structure, good to excellent internal consistency, and high 7-week test–retest reliability. In this study, the CFI also exhibited good internal consistency, with a Cronbach’s alpha coefficient of 0.959.

#### 3.2.3 Physical Activity Self-Efficacy Scale

This study used the Chinese version of the Physical Activity Self-Efficacy Scale (PASE), developed by Motl et al. (2000) and revised by Chen et al. (2019), to measure college students’ beliefs about physical activity (Chen et al., 2019; Motl et al., 2000). The scale contains eight questions, such as “I can exercise most of the week” and “I can exercise even at home.” It is a one-dimensional scale using a 5-point Likert scale (“Strongly Disagree” = 1 to “Strongly Agree” = 5). The total score reflects the college students’ self-efficacy regarding physical activity. A higher total score indicates better self-efficacy. In this study, the Cronbach’s α coefficient of the scale was 0.798.

#### 3.2.4 Physical Activity Rating Scale-3

Sports participation was measured using the Physical Activity Rating Scale (PARS-3) revised by Liang and Lin (1994). The scale comprises three dimensions: frequency, time, and intensity, all self-rated by the participants. Each item was scored from 1 to 5. For descriptive reporting, the published composite score was calculated as intensity × (duration − 1) × frequency (range 0–100), with scores of 19 or lower, 20–42, and 43 or higher conventionally classified as low, moderate, and high activity, respectively. In CFA and SEM, intensity, duration, and frequency were specified as reflective indicators of a latent sports participation factor. Table 1 reports the composite descriptively, whereas the CFA and structural paths refer to the latent factor. Furthermore, the reflective specification was supported in this sample by standardized loadings of 0.676–0.841, composite reliability of 0.813, and average variance extracted of 0.593. Cronbach’s alpha was 0.725.

**Table 1:** Means, standard deviations, and correlations among the main variables.

Variable	M	SD	DHLI	CFI	PASE	PARS-3
DHLI	1.88	0.489	**1**			
CFI	3.865	0.929	0.457***	**1**		
PASE	3.259	0.918	0.232***	0.206***	**1**	
PARS-3	27.190	13.397	0.247***	0.237***	0.201***	**1**

***p < 0.001.

### 3.3 Statistical analysis

Analyses were conducted in SPSS 26.0 and AMOS 26.0. After questionnaire screening, the analytic dataset contained complete responses for all modeled items. No item-level missing values were imputed. Responses on Likert-type items were approximated as continuous variables for estimation. Univariate skewness and kurtosis values were inspected and fell within the prespecified acceptable ranges. Confirmatory factor analysis (CFA) evaluated the study-specific four-factor measurement model, with one first-order latent factor representing each focal construct. Models were estimated by maximum likelihood (MLE). Reliability was summarized with Cronbach’s alpha, and convergent validity with standardized loadings, average variance extracted (AVE), and composite reliability (CR). Discriminant validity was evaluated using the Fornell-Larcker criterion. Pearson correlations were reported descriptively and were not treated as prerequisites for testing indirect associations. The structural equation modeling (SEM) included field of study, region, gender, and grade as covariates of sports participation. Specific indirect associations were evaluated with 5,000 bias-corrected bootstrap resamples and 95% confidence intervals. An interval excluding zero was interpreted as statistical evidence of an indirect association.

## 4 Results

### 4.1 Common method bias diagnostic

Harman’s single-factor diagnostic extracted a first unrotated factor accounting for 28.38% of total variance, below the prespecified 40% reference value. This result indicates that no single factor dominated the item covariance, but the diagnostic cannot establish the absence of common method bias (Podsakoff et al., 2003). Residual bias associated with self-report and common measurement context therefore cannot be ruled out.

### 4.2 Confirmatory factor analysis of digital health literacy, cognitive flexibility, physical activity self-efficacy, and sports participation

Confirmatory factor analysis was performed using AMOS, and the results are shown in Table 2. All fit indices of the four-factor model met the recommended criteria (*χ*^2^ = 1,584.148, df = 1,028, *χ*^2^/df = 1.541, TLI = 0.948, CFI = 0.937, RMSEA = 0.025, SRMR = 0.031), indicating that these four variables have good measurement model fit (See Figure 2). These results support the adequacy of the measurement specification used in this sample. They do not demonstrate that the multidimensional DHLI 2.0 or CFI is inherently unidimensional.

**Table 2:** Confirmatory factor fit indices of digital health literacy, cognitive flexibility, physical activity self-efficacy, and sports participation.

Common indicators	*χ*^2^/df	RMSEA	SRMR	GFI	IFI	TLI	CFI
Standard	<5	<0.08	<0.08	>0.8	>0.9	>0.9	>0.9
Numerical values	1.541	0.025	0.031	0.945	0.967	0.948	0.937

The model contains one study-specific first-order latent factor per focal construct; fit does not establish that the source DHLI 2.0 or CFI is unidimensional.

**Figure 2:**
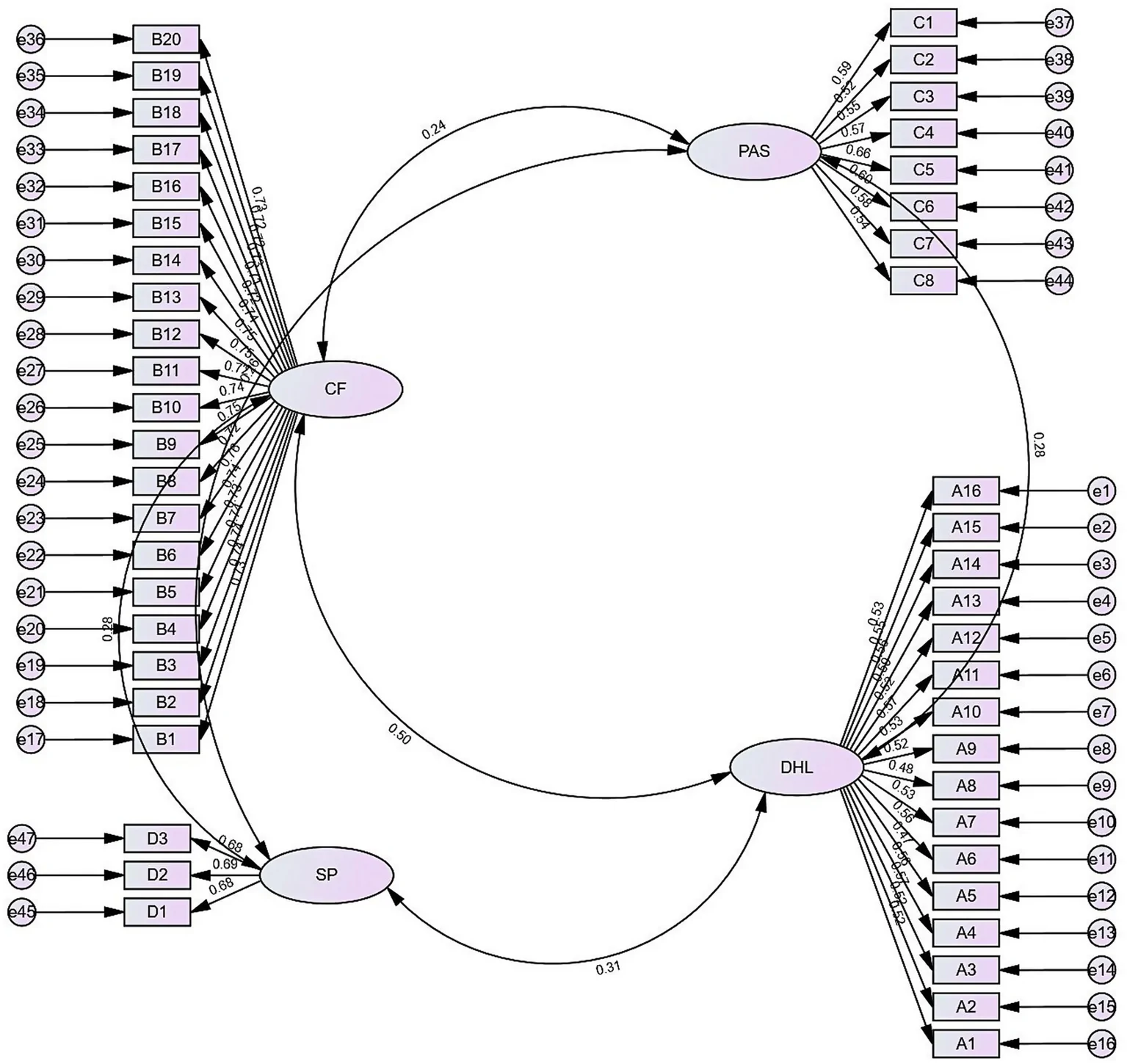
Confirmatory factor analysis of digital health literacy, cognitive flexibility, physical activity self-efficacy, and sports participation.

### 4.3 Convergent validity

Standardized item loadings were 0.656–0.863. For each study-specific latent factor, average variance extracted (AVE) was at least 0.50 and composite reliability (CR) exceeded 0.70, supporting convergent validity for the specified measurement model (See Table 3).

**Table 3:** Standardized loadings, composite reliability, and average variance extracted for the study-specific measurement model.

Variable	Items	Standardized loading	CR	AVE
Digital health literacy	A1	0.821	0.958	0.589
	A2	0.746		
	A3	0.745		
	A4	0.733		
	A5	0.774		
	A6	0.861		
	A7	0.837		
	A8	0.716		
	A9	0.729		
	A10	0.837		
	A11	0.863		
	A12	0.761		
	A13	0.701		
	A14	0.735		
	A15	0.691		
	A16	0.698		
Cognitive flexibility	B1	0.731	0.959	0.539
	B2	0.741		
	B3	0.744		
	B4	0.736		
	B5	0.732		
	B6	0.74		
	B7	0.759		
	B8	0.717		
	B9	0.751		
	B10	0.739		
	B11	0.718		
	B12	0.747		
	B13	0.754		
	B14	0.743		
	B15	0.722		
	B16	0.708		
	B17	0.732		
	B18	0.719		
	B19	0.724		
	B20	0.728		
Physical activity self-efficacy	C1	0.736	0.909	0.557
	C2	0.754		
	C3	0.743		
	C4	0.775		
	C5	0.656		
	C6	0.800		
	C7	0.785		
	C8	0.714		
Sports participation	D1	0.676	0.813	0.593
	D2	0.841		
	D3	0.784		

AVE = average variance extracted; CR = composite reliability. Values refer to the study-specific overarching latent factors used in this analysis.

### 4.4 Discriminant validity

The discriminant validity analysis indicated that all inter-construct correlation coefficients were lower than the square root of the AVE for their respective constructs. These results demonstrate sufficient differentiation among the latent variables, confirming acceptable discriminant validity of the measurement model (See Table 4).

**Table 4:** Fornell-Larcker discriminant-validity assessment.

Variable	Sports participation	Physical activity self-efficacy	Cognitive flexibility	Digital health literacy
Sports participation	0.770			
Physical activity self-efficacy	0.258	0.747		
Cognitive flexibility	0.285	0.237	0.734	
Digital health literacy	0.31	0.276	0.501	0.767

The diagonal line corresponds to the square root of the dimension.

### 4.5 Correlation analysis of variables

Pearson correlations showed positive bivariate associations among all focal variables. Digital health literacy was associated with cognitive flexibility (*r* = 0.457, *p* < 0.001), physical activity self-efficacy (*r* = 0.232, *p* < 0.001), and sports participation (*r* = 0.247, *p* < 0.001). Cognitive flexibility was associated with physical activity self-efficacy (*r* = 0.206, *p* < 0.001) and sports participation (*r* = 0.237, *p* < 0.001), and physical activity self-efficacy was associated with sports participation (*r* = 0.201, *p* < 0.001) (See Table 1).

### 4.6 Analysis of hypothesized indirect associations

Significant correlations were found between digital health literacy, cognitive flexibility, physical activity self-efficacy, and sports participation, meeting the statistical requirements for further mediation effect analysis of cognitive flexibility and self-efficacy. A structural equation model (SEM) was constructed with digital health literacy as the independent variable, sports participation as the dependent variable, and cognitive flexibility and physical activity self-efficacy as intermediate variables. Table 5 and Figure 3 show the overall fit indices, indicating that the proposed path analysis model demonstrated an acceptable overall fit to the data. *χ*^2^/df = 1.984, RMSEA = 0.016, SRMR = 0.033, GFI = 0.930, TLI = 0.993, IFI = 0.982, CFI = 0.986. In summary, these results indicate a high degree of model fit, a reasonable path structure between variables, and data that support the proposed theoretical framework. Gender, grade, region, and subject were entered as covariates in the structural model to account for potential demographic influences on sports participation.

**Table 5:** Fit indices for the hypothesized cross-sectional indirect-association model.

Common indicators	*χ*^2^/df	RMSEA	SRMR	GFI	IFI	TLI	CFI
Standard	<5	<0.08	<0.08	>0.8	>0.9	>0.9	>0.9
Numerical values	1.984	0.016	0.033	0.930	0.982	0.993	0.986

**Figure 3:**
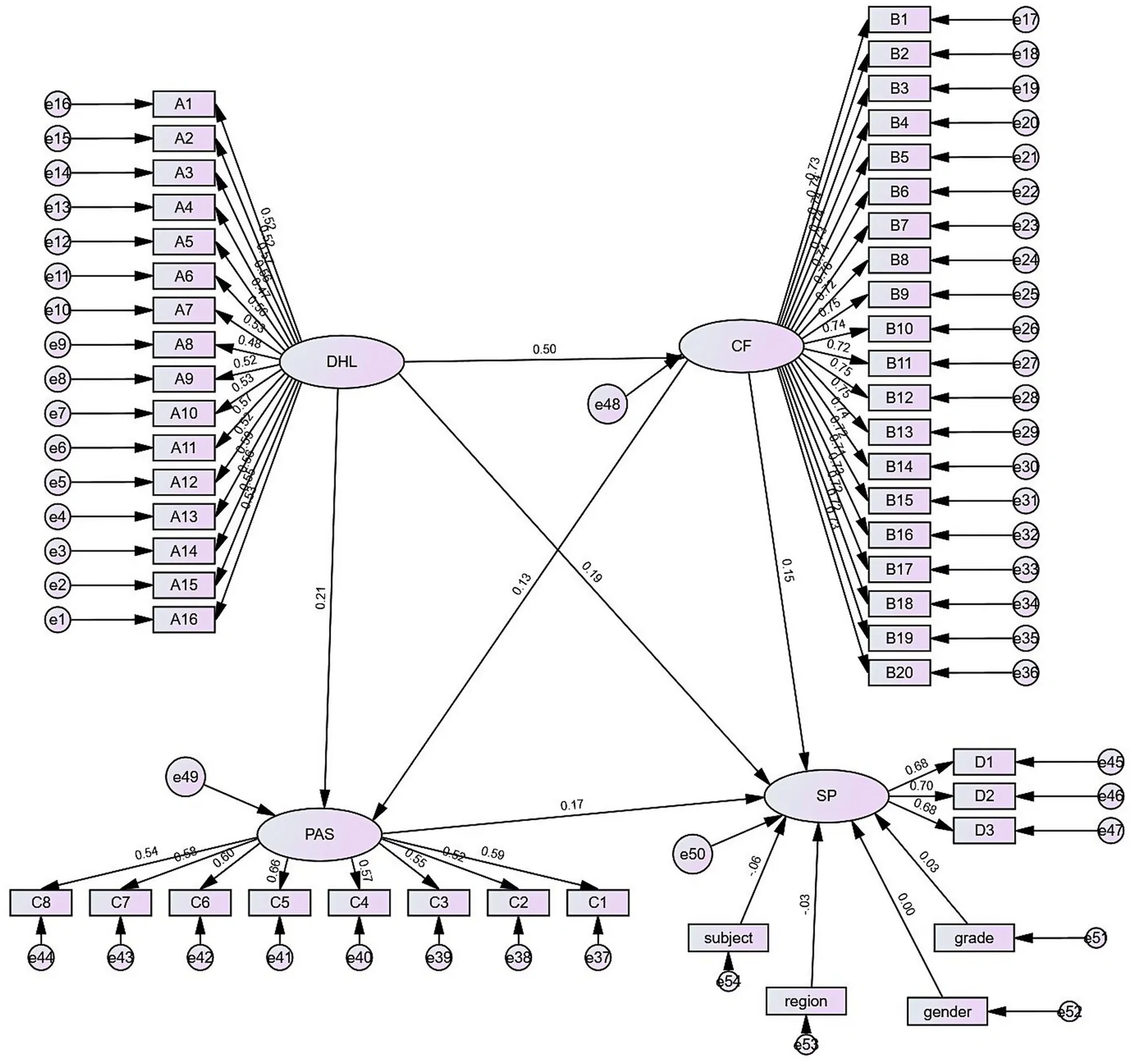
Indirect-association model of cognitive flexibility and physical activity self-efficacy between digital health literacy and sports participation.

### 4.7 Model path coefficient test

Table 6 presents the structural estimates. Digital health literacy was positively associated with cognitive flexibility (*β* = 0.504, *p* < 0.001), physical activity self-efficacy (*β* = 0.213, *p* < 0.001), and sports participation (*β* = 0.191, *p* = 0.002). Cognitive flexibility was associated with physical activity self-efficacy (*β* = 0.134, *p* = 0.012) and sports participation (*β* = 0.150, *p* = 0.003). Physical activity self-efficacy was also associated with sports participation (*β* = 0.173, *p* = 0.002). Except for the digital health literacy-cognitive flexibility association, these standardized coefficients were small. The standardized focal-variable solution corresponded to approximate explained-variance values of *R*^2^ = 0.254 for cognitive flexibility, 0.091 for physical activity self-efficacy, and 0.147 for sports participation. None of the four demographic covariates was statistically associated with sports participation (all *p* > 0.05). The cross-sectional association specified in Hypothesis 1 was supported.

**Table 6:** Structural associations in the hypothesized model.

Path	*β*	B	S.E.	C.R.	*p*
Digital health literacy → Cognitive flexibility	0.504	1.015	0.117	8.982	***
Cognitive flexibility → Self-efficacy	0.134	0.160	0.049	2.438	0.012
Digital health literacy → Self-efficacy	0.213	0.406	0.113	3.53	***
Self-efficacy → Sports participation	0.173	0.215	0.069	3.083	0.002
Digital health literacy → Sports participation	0.191	0.447	0.146	3.063	0.002
Cognitive flexibility → Sports participation	0.150	0.184	0.064	2.697	0.003
Subject → Sports participation	−0.067	−0.097	0.059	−1.350	0.147
Region → Sports participation	−0.034	−0.075	0.094	−0.514	0.513
Gender → Sports participation	−0.041	−0.064	0.094	−0.091	0.889
Grade → Sports participation	0.036	0.053	0.042	0.763	0.421

*β* = standardized estimate; *B* = unstandardized estimate; C.R. = critical ratio. ***p < 0.001.

### 4.8 Bootstrap indirect-association estimates

Table 7 reports standardized specific indirect associations based on 5,000 bias-corrected bootstrap resamples and 95% confidence intervals. The estimates quantify how contemporaneous covariance was partitioned under the specified model, and they do not establish a process unfolding over time.

**Table 7:** Bias-corrected bootstrap estimates of cross-sectional indirect associations.

Path	Effect	Lower	Upper	*p*
Digital health literacy → Cognitive flexibility → Sports participation	0.076	0.018	0.145	0.01
Digital health literacy → Physical activity self-efficacy → Sports participation	0.036	0.008	0.084	0.006
Digital health literacy → Cognitive flexibility → Physical activity self-efficacy → Sports participation	0.011	0.002	0.031	0.012
Total effect	0.312	0.191	0.412	0.001
Direct effect	0.189	0.038	0.322	0.014
Indirect effect	0.123	0.059	0.214	0.001

The specific indirect association between digital health literacy and sports participation through cognitive flexibility was 0.076 (95% CI [0.018, 0.145], *p* = 0.010). Because the interval excluded zero, the cross-sectional pattern was consistent with Hypothesis 2.

The specific indirect association through physical activity self-efficacy was 0.036 (95% CI [0.008, 0.084], *p* = 0.006). The interval excluded zero, providing cross-sectional statistical support for Hypothesis 3.

The theory-ordered sequential indirect association through cognitive flexibility and physical activity self-efficacy was 0.011 (95% CI [0.002, 0.031], *p* = 0.012). Although statistically different from zero, its magnitude was small. The covariance pattern was consistent with Hypothesis 4.

## 5 Discussion

Digital health literacy represents an important psychological resource in digitally mediated health environments. This study examined whether digital health literacy was associated with sports participation and whether the covariance pattern was consistent with prespecified indirect links through cognitive flexibility and physical activity self-efficacy. Digital health literacy showed a direct standardized association of 0.191 with sports participation, and the total indirect association was 0.123. The sequential component was only 0.011. Thus, the results support a statistically coherent cross-sectional association model, not evidence that a psychological process unfolded in the proposed order. A noteworthy contribution of the present study is that it extends the understanding of physical activity behavior from traditional motivational explanations toward a digital cognitive perspective. While previous studies have primarily focused on motivational determinants of sports participation, the current findings suggest that adaptive information-processing abilities may represent an additional psychological mechanism through which digital competencies are associated with health behavior.

Digital health literacy showed a significant positive association with sports participation. This finding aligns with previous studies indicating that individuals with higher levels of digital health literacy are more likely to engage in health-promoting behaviors, including regular physical activity (Jiang et al., 2024; Shudayfat et al., 2023). In the current digital environment, college students increasingly rely on social media, health applications, wearable devices, and online exercise platforms to obtain exercise-related information. Individuals with stronger digital health literacy are generally more capable of identifying reliable health information, filtering misinformation, and transforming fragmented digital resources into actionable health behaviors. Unlike traditional health literacy, digital health literacy emphasizes not only comprehension ability but also interactive information processing and decision-making capacity within technology-based environments (Norman and Skinner, 2006). Therefore, students with higher digital health literacy may be more willing to seek exercise knowledge and maintain participation in sports activities actively.

The specific indirect association through cognitive flexibility (0.076) was larger than the indirect association through physical activity self-efficacy alone (0.036), although both were small in absolute terms. In digitally saturated environments, cognitive resources may represent a potentially important psychological factor associated with sports participation in digital environments. Unlike traditional exercise behavior models that primarily emphasize motivation or behavioral intention, the current findings highlight the importance of adaptive cognitive processing under conditions characterized by information fragmentation, multitasking, and continuous digital stimulation. This may help explain why students with stronger cognitive flexibility were more likely to maintain higher levels of sports participation despite the unstable and distraction-rich nature of contemporary digital environments (Dennis and Vander Wal, 2010). Previous studies have shown that cognitively flexible individuals demonstrate better emotional regulation, behavioral adaptation, and problem-solving ability under stress (Nakhostin-Khayyat et al., 2024; Yurt and Hayli, 2025).

Physical activity self-efficacy was positively associated with sports participation (*β* = 0.173), consistent with social cognitive theory’s emphasis on efficacy beliefs (Bandura, 2013). Whether college students maintain a regular exercise routine depends not only on knowledge acquisition but also on their belief that they can effectively maintain exercise amidst their busy daily lives. In today’s digital environment, exercise-related apps, wearable devices, and online fitness communities can continuously provide behavioral feedback, social comparisons, and short-term achievement experiences. These experiences can gradually enhance students’ confidence in managing exercise behavior under pressure. Therefore, digital health literacy can indirectly promote participation in physical activity by improving individuals’ ability to organize and maintain physical activity in daily life (Bebeley et al., 2017; Dishman et al., 2005). The current findings further extend this literature by demonstrating that digital health literacy may serve as an upstream psychological resource that contributes to the formation of self-efficacy.

Students who possess stronger digital health literacy may first become more cognitively adaptive when dealing with exercise-related information and lifestyle challenges. This increased flexibility may subsequently strengthen their confidence in maintaining physical activity, ultimately contributing to more stable sports participation. Compared with traditional linear explanations of exercise behavior, the present findings emphasize that sports participation in digital environments may involve multiple interconnected psychological adjustment processes rather than a single motivational factor alone (Mohammadkhani et al., 2022; Woo et al., 2024). The indirect association between digital health literacy, cognitive flexibility, physical activity self-efficacy, and sports participation was statistically significant, but its value was only 0.011, indicating a weak association with potentially limited practical significance. Theoretically, students’ ability to access and use digital health information may be related to their ability to flexibly adjust their thinking, and cognitive flexibility may be related to confidence in physical activity self-efficacy, which in turn is related to sports participation. This interpretation is consistent with the overall association pattern of the research data. However, since all variables were measured at the same time, this study cannot prove that these variables occurred in the above order, nor can it rule out the possibility of inverse relationships or mutual influences. Future studies should employ longitudinal research with longer time intervals to further examine whether changes in digital health literacy precede changes in cognitive flexibility, physical activity self-efficacy, and sports participation.

In conclusion, digital health literacy may play a broader role in college students’ health behaviors than previously thought. Digital health literacy is not merely the technical ability to search for health information online. It may also influence how individuals cognitively adapt to complex digital environments and maintain confidence in long-term behavioral regulation. Against the backdrop of the rapid development of digital fitness culture and technology-driven lifestyles, understanding these psychological pathways may become increasingly important for future strategies to promote physical activity among young people.

## 6 Conclusion

Among 600 college students from one Chinese university, digital health literacy was positively associated with cognitive flexibility, physical activity self-efficacy, and sports participation. The prespecified indirect-association model fit the data, but most coefficients were small, and the ordered three-path estimate was only 0.011. The study therefore identifies a theory-consistent covariance pattern rather than a verified causal mechanism. Its main contribution is to connect digital-health-information competence with adaptive cognition and exercise-related beliefs while making clear that temporal ordering and practical effects require longitudinal, experimental, and multi-institutional confirmation.

## 7 Limitations and future directions

Digital health literacy may simultaneously function as both a health resource and a potential cognitive burden. First, the cross-sectional design cannot establish temporal precedence, causal direction, or longitudinal mediation; reverse and reciprocal relations are plausible (Maxwell and Cole, 2007). Future studies should use at least three temporally separated assessments or experimental manipulation when testing the proposed sequence. Second, while higher digital health literacy may facilitate sports participation through improved information processing and behavioral regulation, excessive exposure to digital information may also contribute to information overload, screen fatigue, attentional fragmentation, and digital dependence. These negative aspects of digital environments have received comparatively limited attention in existing physical activity research. Therefore, future studies should adopt a more balanced perspective by examining both the beneficial and potentially adverse psychological consequences of digital health environments. Third, all variables were measured using self-report questionnaires, which may increase the possibility of response bias and social desirability effects. Although Harman’s single-factor test suggested that common method bias was not severe, the use of a single data collection approach may still influence the stability of the findings. Future studies may benefit from combining objective indicators, such as wearable device data, exercise records, or digital behavior tracking, to improve measurement accuracy and ecological validity. Finally, the current sample was limited to college students, which may reduce the generalizability of the findings to other populations. Digital health literacy and exercise behavior may differ substantially across age groups, educational backgrounds, and sociocultural contexts. Future research should examine whether the proposed psychological mechanisms remain stable among adolescents, older adults, athletes, or clinical populations.

## Data Availability

The original contributions presented in the study are included in the article/supplementary material, further inquiries can be directed to the corresponding author/s.
